# High-performance MnO_2_-deposited graphene/activated carbon film electrodes for flexible solid-state supercapacitor

**DOI:** 10.1038/s41598-017-11267-0

**Published:** 2017-10-09

**Authors:** Lanshu Xu, Mengying Jia, Yue Li, Xiaojuan Jin, Fan Zhang

**Affiliations:** MOE Engineering Research Center of Forestry Biomass Materials and Bioenergy, Beijing Key Laboratory of Lignocellulosic Chemistry, Beijing Forestry University, Beijing, 100083 China

## Abstract

High theoretical capacitance of MnO_2_ nanoparticles were successfully electrodeposited on the conductive graphene/activated carbon (GN/AC) composite film, and the urchin type MnO_2_ microspheres were controlled by adjusting the electro-deposition reaction times. The GN/AC/MnO_2_-1200s composite electrodes exhibited a maximum specific capacitance of 1231 mF/cm^2^ (MnO_2_ loading mass of 7.65 mg/cm^2^ and the mass specific capacitance of 123 F/g) at a current density of 0.5 mA/cm^2^. The assembled flexible solid-state symmetric supercapacitor had a good mechanical flexibility (about 88.6% of its original capacitance after 500 bending times) and prominent cycling stability (about 82.8% retention in capacitance over 10000 cycles). More importantly, the device could possess a maximum energy density of 0.27 mW h/cm^3^ and a maximum power density of 0.02 W/cm^3^. These results well demonstrate a great potential for applications of GN/AC/MnO_2_ composite electrodes in flexible energy storage devices.

## Introduction

Graphene (GN), an atom-thick, honeycomb two-dimensional structure, has the characteristics of higher specific surface area, excellent conductivity or mechanical flexibility^1–3^. It has attracted a great deal of concerns and is widely used in flexible composite electrodes^4–7^. However, the preparation process of graphene is extremely complex, high-cost and especially prone to agglomeration or stacking, which seriously affect the infiltration of the electrolyte and reduce the utilization ratio of specific surface area^8^. Activated Carbon (AC), as the earliest and the most widely application of electrode materials in supercapacitors (SCs), possesses the advantages of larger specific surface area, higher electrochemical stability, low price, environmental friendliness, rich raw materials and renewable, etc^9–11^. The composition of GN/AC can be applied in the field of flexible electrodes and achieve a significant synergistic effect. The two-dimensional flake GNs serve as adhesive to bond ACs together by self-assembly method, which made the composite electrodes have good mechanical flexibility and provide a conductive bridge for ion transport. Meanwhile, the doping of AC expands the layer spacing and can prevent the agglomeration or stacking of GN in some extent. Moreover, the formed three-dimensional (3D) porous structure also can increase the specific surface area and improve the electrical conductivity. AC is a promising partial substitute for GN in flexible electrodes.

Manganese oxide materials are widely known as the pseudocapacitance based on reversible redox reactions at the surface of active materials^9,12^. Compared with other metal oxides, MnO_2_ electrodes have significant advantages of low price, high theoretical specific capacitance and environmental compatibility^13–15^. However, there are still significant drawbacks of MnO_2_ electrodes. Namely, the low electronic conductivity leads to relative low power density, and the low cycling life due to the disproportionate dissolution reaction of MnO_2_ active material, which results in fast performance fading during cycling^16–18^. The key to solve these problems is to explore a novel flexible electrode material system containing the material combination, material morphology and distribution, and choice of electrolytes that even under high voltages, high electrical conductivity and electrochemical stability can be guaranteed^19,20^. In recent years, researches have explored many possible routes using different flexible electrode materials, such as carbon nanotubes (CNTs)^21^, reduced graphene oxide (rGO)^22^, rGO/CNTs^23^, MnO_2_/rGO^24^ and so on. For example, CNT supercapacitor showed an energy density of 0.601 mWh/cm^3^ and rGO supercapacitor displayed a value of 0.17 μWh/cm^3^. The energy density could be generally increased by well designing the composite structures. An emerged strategy of combining renewable carbon-based materials with pseudocapacitive MnO_2_ can achieve cost and environmental advantages, high electrochemical performance, and long cycle life, benefiting from both mechanisms of double-layer supercapacitor and pseudocapacitor^25–28^.

In this work, a flexible and binder-free ternary GN/AC/MnO_2_ composite electrode film was successfully synthesized by facile vacuum filtration and electro-deposition processes. The porous AC materials were interspersed between GN sheets as electrode substrates, facilitating the electrolyte ion transport and the deposition of MnO_2_. The effects of MnO_2_ morphology on the GN/AC composite films were controlled by simply adjusting the reaction times and the possible deposition mechanism of MnO_2_was derived. Moreover, the electrochemical performances and mechanical property were systematically studied. As expected, the measurement results indicated that the MnO_2_ morphology greatly affected the electrochemical performance of GN/AC/MnO_2_ composite electrodes, and the flexible ternary electrode exhibited a high specific capacitance of 1231 mF/cm^2^ at a current density of 0.5 mA/cm^2^. Furthermore, the as-assembled flexible solid-state SCs showed a stable electrochemical performance and higher energy density.

## Results

**Figure 1:**
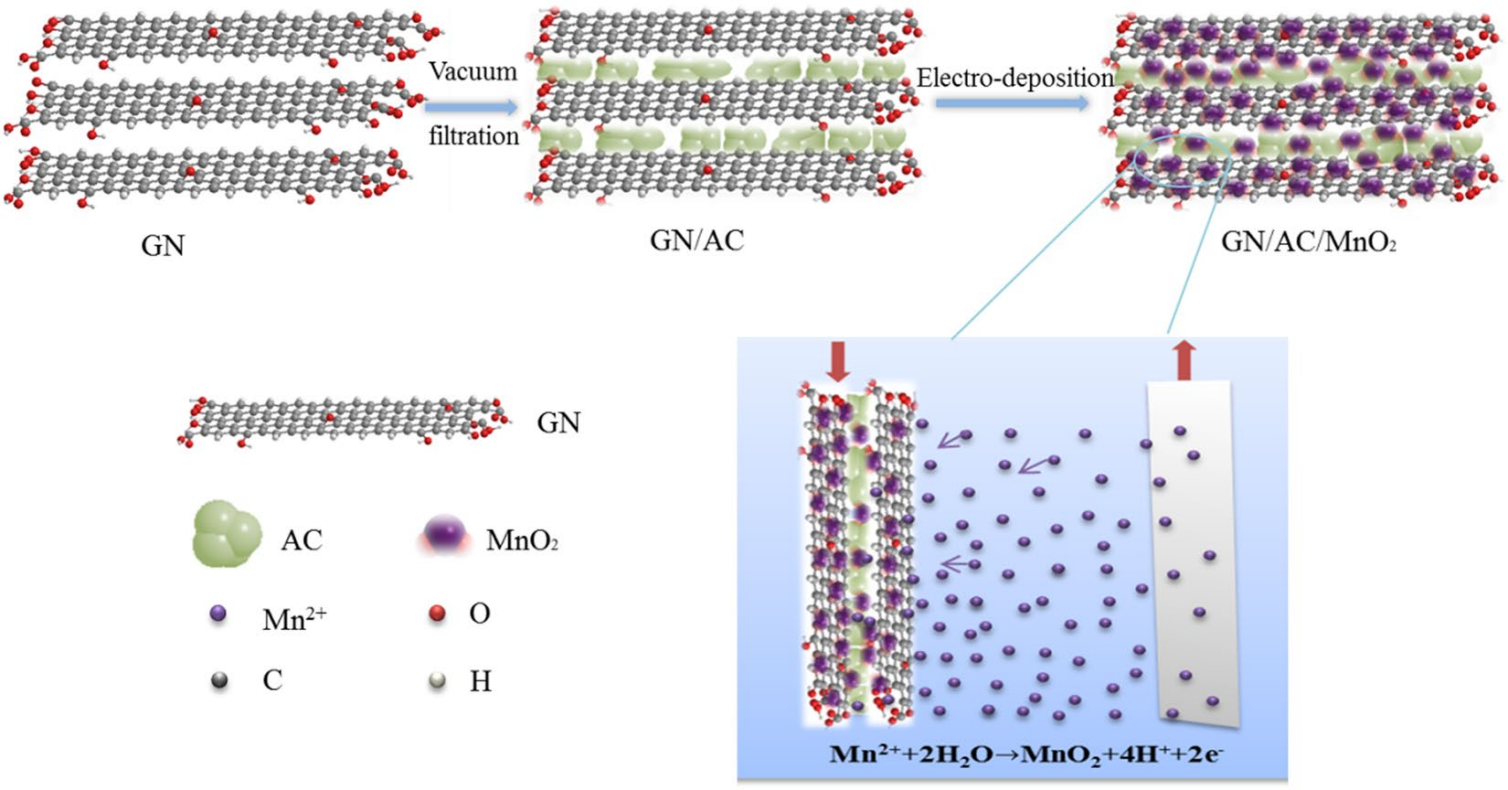
Schematic illustrations for the preparation process of GN/AC/MnO2 composite electrodes.

GN/AC/MnO_2_ composite electrodes were prepared by two steps (Fig. 1): firstly, AC particles were bonded together with two-dimensional GN sheets using self-assembly method by a facile vacuum filtration process, which expanded the layer spacing and increased the specific surface area of GN. Secondly, the resulted GN/AC flexible films were used as the working electrode and MnO_2_ nanostructures were electrodeposited on the surface of GN/AC flexible films. The formation mechanism can be summarized briefly. GN/AC flexible films were immersed into a plating solution containing Mn(CH_3_COO)_2_ and Na_2_SO_4_. In anodic constant current electro-deposition process, the charged ions in the electrolyte were directed diffusion under an applied electric field, low valence metal ions were discharged near the anode and the high valence ions were formed, leading to Mn (II) was oxidized into Mn (IV). The redox reaction occurred on the electrode surface: Mn^2+^ + 2H_2_O $\to$MnO_2_ + 4 H^+^ + 2e^−^.

**Figure 2:**
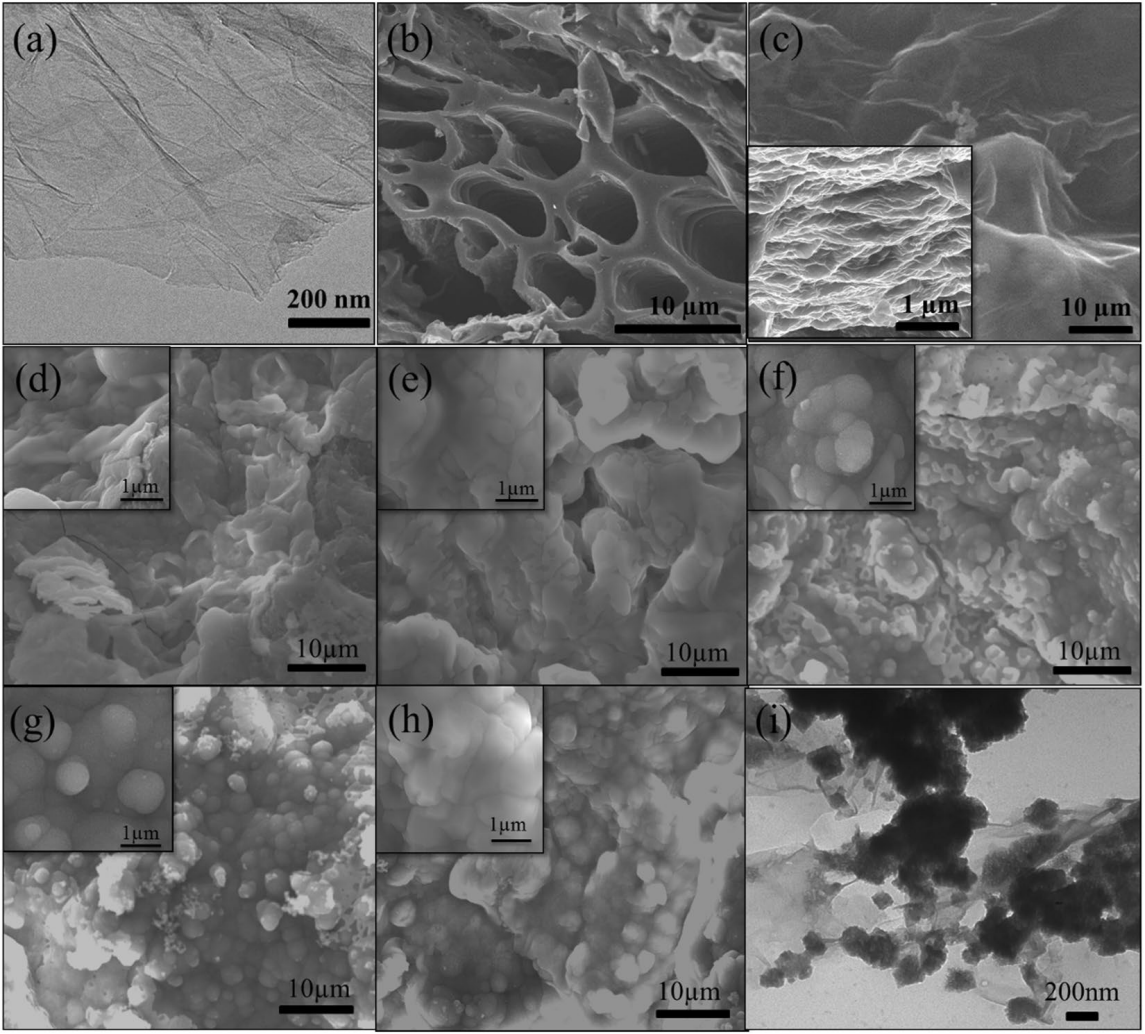
(**a**) TEM image of GN sheet; (**b**) FESEM image of ACs; (**c**) SEM image of flexible GN/AC substrate; SEM images of GN/AC/MnO2 composite films prepared at various reaction times: (**d**) 300 s, (**e**) 600s, (**f**) 900s, (**g**) 1200s and (**h**) 1500s; (**i**) TEM image of GN/AC/MnO2-1200s composite films.

**Figure 3:**
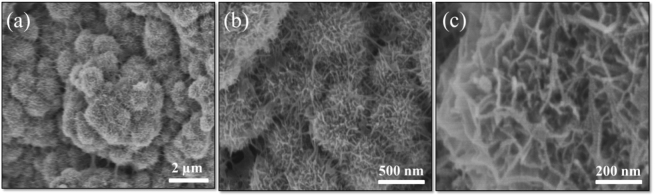
GN/AC/MnO2-1200s composite films at high magnification.

Figure 2a showed the TEM image of GN sheets. The layered graphene tended to stack together to form agglomerate due to the chemical inertness of reduced graphene oxide^29^. From that we can see the obtained GN was very well dispersed as a single-layer sheet. The porous ACs can be seen in Fig. 2b, the highly porous morphology was beneficial to the transmission of electrolyte ions. The SEM images (Fig. 2c) of the GN/AC flexible film showed that the GN sheets resembled adhesive to connect ACs together and provided a conductive bridge for ion transport. Obviously, the porous carbon materials and the formed wavy surface increased the specific surface area of GN. Also can be seen from the cross-section image (insert in Fig. 2c), the AC particles were interspersed in GN layers, which extended the interlayer spacing and prevented the flocculation and accumulation of GN sheets to some certain extent. The extended GN/AC flexible film was conducive to the deposition of MnO_2_. Figure 2d–h showed the growth of MnO_2_ microspheres on GN/AC flexible films in the electro-deposition process with various reaction times of 300 s, 600 s, 900 s, 1200 s and 1500 s. As shown in Fig. 2d, small MnO_2_ nanoparticles were deposited on the film surface when the reaction time was 300 s. A dense MnO_2_ nanoparticle layers were formed on the GN/AC flexible films with the reaction time increased to 600 s (Fig. 2e). The uniform structure of GN/AC films with strong adhesion and good mechanical property could provide a large surface area, therefore, a higher number of active sites, leading to extensive coverage of MnO_2_ nanoparticles. Further increasing the reaction time from 600 s to 1200 s, the morphology and structure of GN/AC/MnO_2_ composite films were significantly changed. A large quantity of MnO_2_ microspheres were gradually formed and observed on the surface (Fig. 2f,g). The electric potential continued to increase with further increase of reaction time at the current density of 8 mA/cm^2^. The higher electric potential might be conducive to nucleation and negatively inhibit crystal growth. When the deposition time reached 1500 s (Fig. 2h), more MnO_2_ microspheres generated and stacked together, the accumulation of MnO_2_ nanoparticles layer might cause the film too dense (~8.25 mg/cm^2^ mass loading), which decreased the specific surface area and was not conducive to ion transport. The TEM image of GN/AC/MnO_2_-1200s was showed in Fig. 2i, it can be observed that MnO_2_ microspheres were uniformly dispersed in flexible substrate. In addition, the AC/MnO_2_ attached to the surface of GN forming a distinct obstacle between GN layers, increasing the specific surface area, which is beneficial to improve the electrochemical properties. Moreover, high-magnification FESEM images of GN/AC/MnO_2_-1200s were displayed in Fig. 3a,b and c. The formed MnO_2_ microspheres, similar to urchins, have a diameter of 150–300 nm. Obviously, the formation of urchin type MnO_2_ microspheres dramatically increased the specific surface area of the active materials, which benefited to the infiltration and transmission of the electrolyte. Moreover, the urchin type MnO_2_ microspheres can be easily controlled by simply adjusting the electro-deposition reaction times.

**Figure 4:**
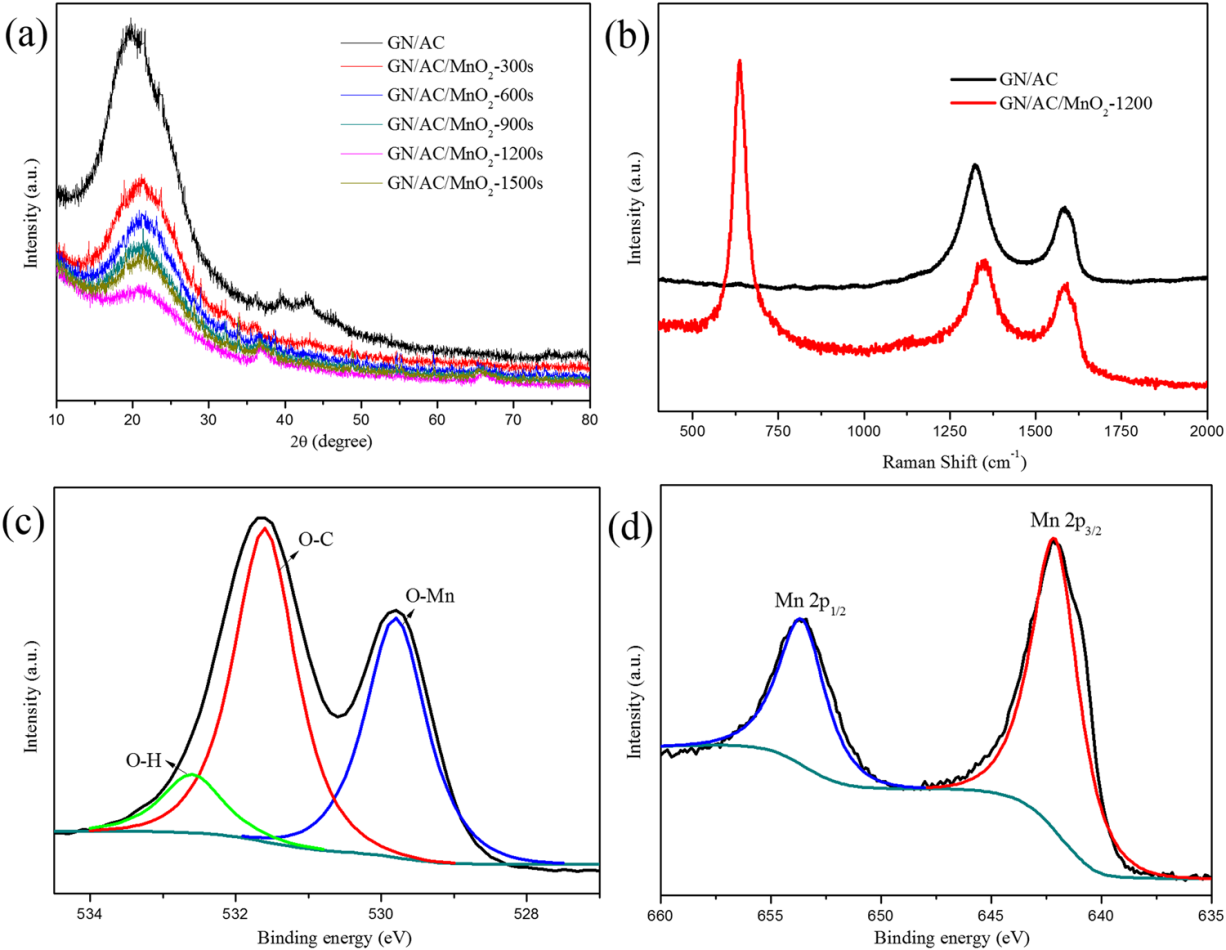
(**a**) XRD patterns, (**b**) Raman spectra, XPS spectra (**c**) O 1s, (**d**) Mn 2p.

The X-ray diffraction (XRD) for GN/AC and GN/AC/MnO_2_-1200s were further analyzed. As shown in Fig. 4a, two diffraction peaks (2Ѳ) was observed at around 22° and 43° for the GN/AC composites, corresponding to the (002) and (101) crystal planes, respectively, which are the characteristic peaks of carbon-based material^30,31^. With the increase of MnO_2_ deposition, the intensity of carbon-based characteristic peaks decreased accordingly, especially for GN/AC/MnO_2_-1200s composite film. Moreover, two sharp peaks around 37° and 66° appeared for GN/AC/MnO_2_ composite films, which could be indexed to the birnessite-type MnO_2_ (JCPDS 42-1317)^32,33^. To further elucidate the detailed structures and chemical compositions of MnO_2_ microspheres on GN/AC (1200 s) films, Raman spectra and X-ray photoelectron spectroscopy (XPS) were probed, and the results were shown in Fig. 4b and Fig. 4c,d. As presented in Fig. 4b, the GN/AC flexible films exhibited two obvious peaks around 1320 cm^−1^ and 1580 cm^−1^, corresponding to the D band and G band of carbon-based material, respectively. After the deposition of MnO_2_ on GN/AC film for 1200 s, a sharp peak around 635 cm^−1^ appeared, which could be assigned to the (O-Mn) stretching vibration of divalent Mn ions, and the broad peak might be ascribed to the overlap of various characteristic peaks from MnO_2_, Mn_3_O_4_ and MnOOH^9,34^. Moreover, the I_D_/I_G_ ratio of GN/AC films increased to 1.22 from 1.21 after electro-deposited MnO_2_, indicating that the deposition of MnO_2_ on GN/AC films had a slight increase of defect ratio and a negligible effect on the structure of the substrate. Typical XPS spectra of O 1 s and Mn 2p for MnO_2_ electro-deposited GN/AC at 1200 s were shown in Fig. 4c,d. From the Fig. 4c, we can see that three peaks at around 529.8, 531.6 and 532.6 eV in the O 1 s spectra, which corresponded to the O-Mn, O-C, and O-H bonds, respectively^35^. The high-resolution Mn 2p spectra was presented in Fig. 4d, from which can be seen that two binding energies were centered at 642.1 eV and 653.7 eV, corresponding to Mn 2p_3/2_ and 2p_1/2_ peak, respectively, with a spin energy separation of 11.6 eV, which revealed a mean valence state of 4+ for Mn in MnO_2_ and matched well with previously reported^36,37^. Furthermore, the result was consistent with XRD analysis as mentioned above.

**Figure 5:**
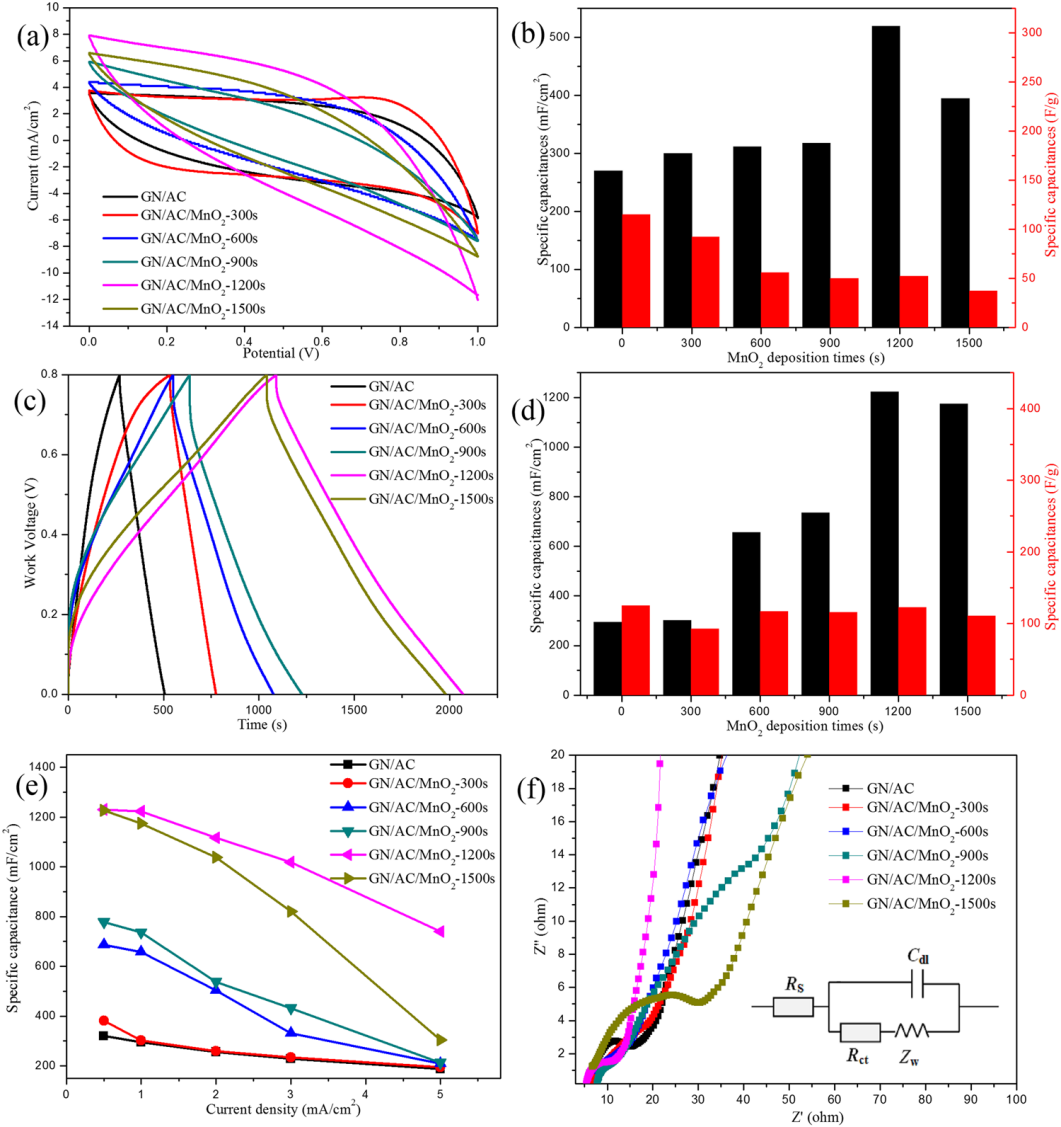
(**a**) CV curves of all samples at the scan rate of 10 mV/s; (**b**) Specific capacitances of all samples at 10 mV/s under the different deposition times; (**c**) GCD curves of all samples at the current density of 1 mA/cm2; (**d**) Specific capacitances of all samples at 1 mA/cm2 under the different deposition times; (**e**) Specific capacitances of all samples at various current densities; (**f**) Nyquist plot of all samples.

The electrochemical performances of the GN/AC/MnO_2_ composite electrodes were firstly evaluated by CV and GCD curves using a three-electrode system in 1.0 M Na_2_SO_4_ aqueous electrolyte. Figure 5a compared the CV curves of the GN/AC/MnO_2_ composite electrodes with various MnO_2_ deposition times ranging from 0 to 1500 s in a potential window of 0–1.0 V at the same scan rate of 10 mV/s. The ternary composites of GN/AC/MnO_2_ have a larger enclosed area than binary composite of GN/AC, indicating that MnO_2_-deposited on the GN/AC flexible films greatly enhanced the electrochemical performance due to the synergistic effects. The addition of ACs expanded the layer spacing of GN sheets, facilitating the deposition of MnO_2_, which was beneficial to enhance the electrochemical properties. The specific capacitance can be calculated from CV curves based on Equation (1) and plotted in Fig. 5b. The area specific capacitances of GN/AC/MnO_2_-1200s electrodes (520 mF/cm^2^ at a scan rate of 10 mV/s) were much larger than others, and then it decreased slightly when the deposition time increased to 1500 s. Moreover, as the amount of MnO_2_ deposition increases, the mass specific capacitances of the composite electrodes are going to decrease accordingly. But for applications such as flexible and small energy storage devices, the amount of energy stored per area is more important than energy per mass^38^. Figure 5c showed GCD curves of all samples at a current density of 1 mA/cm^2^. The triangular shapes were slightly deformed, indicating that the capacitance came from the combination of both electrochemical double-layer capacitance and pseudocapacitance^39^. Similar to the CV curves, the GN/AC/MnO_2_-1200s possessed the longest discharge time than others. According to the Equation (2), the specific capacitances with different deposition times were calculated and plotted in Fig. 5d. For the GN/AC, GN/AC/MnO_2_-300, 600, 900, 1200 and 1500 s composite electrodes, the corresponding specific capacitances were 295 (125), 302 (93), 658 (117), 736 (116), 1224 (122), 1175 mF/cm^2^ (111 F/g), respectively. It should be noted that GN/AC/MnO_2_-1200s has a higher specific capacitance of 1224 mF/cm^2^ at 1 mA/cm^2^, and the mass specific capacitance was around 122 F/g with a mass of 0.01 g/cm^2^ (MnO_2_ loading mass of 7.65 mg/cm^2^). Moreover, It is worth pointing out that such mass loading of MnO_2_ is much higher than other reported literatures^40^, indicating a much larger surface area of GN/AC flexible films^41^. Based on the above electrochemical measurements, the results could be proved that the optimized electro-deposition time was 1200 s for GN/AC/MnO_2_ electrodes. Furthermore, the area specific capacitances of all samples calculated by GCD curves were showed in Fig. 5e. Obviously, GN/AC/MnO_2_-1200s composite electrodes had a dramatic specific capacitance of 1231 mF/cm^2^ (mass specific capacitance of 123 F/g) at 0.5 mA/cm^2^, more than 3.85 times higher than GN/AC flexible films (320 mF/cm^2^). Upon increasing the current density up to 5 mA/cm^2^, the specific capacitance of GN/AC/MnO_2_-1200s composite electrodes (740 mF/cm^2^) could retain about 60% of its original values, and more than 3.98 times higher than GN/AC flexible films (186 mF/cm^2^). For GN/AC/MnO_2_-1500s, the specific capacitance decreased prominently from 1227 mF/cm^2^ at 0.5 mA/cm^2^ to 304 mF/cm^2^ at 5 mA/cm^2^, which only retained about 25%. The decrease of the specific capacitance was ascribed to the excessive deposition time, which might cause the film too compact and deteriorate the ion transport. Further demonstration can be seen in Fig. 5f, the GN/AC/MnO_2_-1500s electrode had a large resistivity (${R}_{s}$, ~21.11 Ω), which was not benefit for electron transport. The Nyquist plot of GN/AC/MnO_2_-1200s composite started from the *Z*′-axis and progressed almost vertically to the *Z″*-axis at the low frequency, indicating that the ideal capacitive characteristics of the electrode^42^.

**Figure 6:**
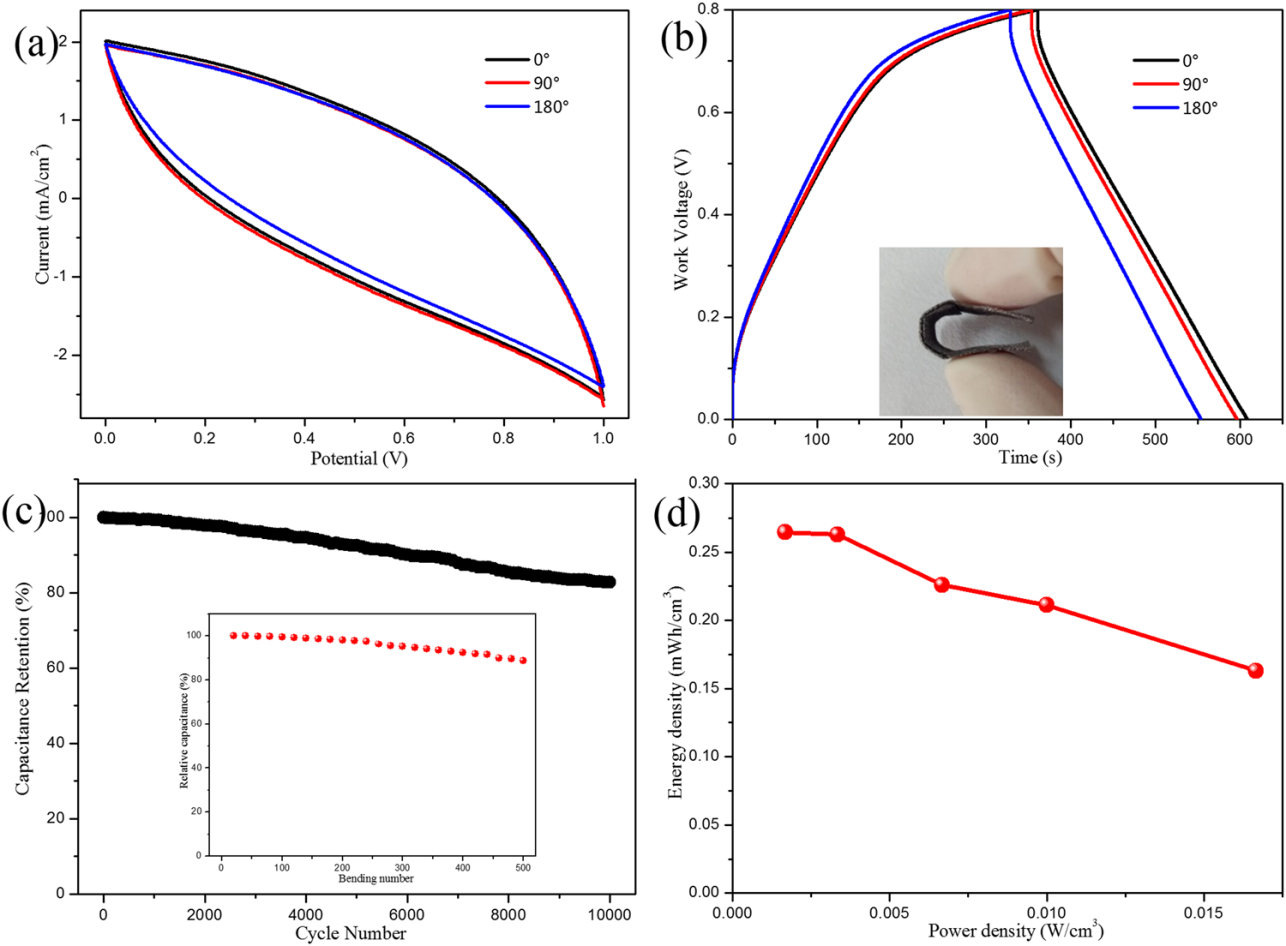
(**a**) CV curves of the flexible devices at 10 mV/s under different bending angles; (**b**) GCD curves of the flexible devices at 1 mA/cm2 under different bending angles; (**c**) Cycling stability of the flexible devices over 10000 cycles and the inset is the mechanical flexibility under different bending times; (**d**) Energy density as a function of power density.

To explore the superior performances of 3D GN/AC/MnO_2_ electrodes in flexible energy storage devices, symmetric flexible solid-state SCs have been assembled by fixing two GN/AC/MnO_2_-1200s electrodes in parallel with PVA/Na_2_SO_4_ electrolyte. As can be seen from Fig. 6a and b, CV curves and GCD curves were nearly overlapped when the bending angles of the flexible device varied from 0° to 180°. And only about 10% fading for the specific capacitance when bended to 180°, demonstrating the electrodes had negligible influence on folding or bending. Using Equation (3), the volumetric capacitance of flexible GN/AC/MnO_2_ device was 2.96 F/cm^3^ at 1 mA/cm^2^. Evidently, the volumetric capacitance is considerably superior to recently reported devices, such as H-TiO_2_@MnO_2_//H-TiO_2_@C (0.70 F/cm^3^ at 0.5 mA/cm^2^)^37^, RGO//MnO_2_ (0.75 F/cm^3^ at 10 mV/s)^40^, MnO_2_//Fe_2_O_3_ (1.5 F/cm^3^ at 2 mA/cm^2^)^43^, MnO_2_//Fe_2_O_3_ (1.2 F/cm^3^ at 10 mV/s)^44^, MnO_2_//Ti-Fe_2_O_3_@PEDOT (2.40 F/cm^3^ at 1 mA/cm^2^)^45^. For practical application, the cycling stability and mechanical flexibility were conducted. As shown in Fig. 6c, approximately 82.8% retention in capacitance over 10000 cycles and the capacitance still maintained at 88.6% of its original value after 500 bending times at 5 mA/cm^2^, indicating the device had an excellent stability and good mechanical flexibility. Furthermore, according to Equations (4) and (5), the calculated energy densities *E* and power densities *P* of our flexible SCs are shown in Fig. 6d. The as-assembled device can possess a maximum energy density of 0.27 mWh/cm^3^ at 0.5 mA/cm^2^ and a maximum power density of 0.02 W/cm^3^ at 5 mA/cm^2^. Therefore, we can determine that the flexible GN/AC/MnO_2_ SCs, with a good mechanical flexibility and superior electrochemical performance, are promising candidates for assembling flexible, environment friendly, low price and ultrathin SCs.

## Discussion

In summary, we reported a two-step process for constructing a 3D GN/AC/MnO_2_ flexible electrode using vacuum filtration and electro-deposition methods. The synergistic effects among 2D graphene sheets, porous activated carbon and high theoretical capacitance of MnO_2_ conduced to obtain excellent electrochemical performance. The 3D GN/AC substrate facilitated the growth of MnO_2_ and enhanced the conductivity. The urchin type MnO_2_ microspheres could be simply controlled by adjusting the electro-deposition reaction times. The GN/AC/MnO_2_-1200s composite electrodes exhibited a maximum specific capacitance of 1231 mF/cm^2^ with a mass of 0.01 g/cm^2^ (MnO_2_ loading mass of 7.65 mg/cm^2^) at a current density of 0.5 mA/cm^2^. Furthermore, approximately 82.8% retention in capacitance over 10000 cycles and the capacitance still maintained at 88.6% of its original value after 500 bending times. In addition, the as-assembled device could possess a maximum energy density of 0.27 mFmWh/cm^3^ at 0.5 mA/cm^2^ and a maximum power density of 0.02 W/cm^3^ at 5 mA/cm^2^. These results well prove that our GN/AC/MnO_2_ composite electrodes have broad prospects in flexible and wearable electronic products.

## Experimental

### Chemicals and materials

Graphite powers were purchased from Alfa Aesar and waste fiberboard materials were used to product ACs. Sodium nitrate (NaNO_3_), sulfuric acid (H_2_SO_4_), potassium permanganate (KMnO_4_), hydrochloric acid (HCl), manganese(II) acetate tetrahydrate [Mn(CH_3_COO)_2_·4H_2_O] and sodium sulfate (Na_2_SO_4_) were obtained from Sinopharm Chemical Reagent Co., Ltd (Beijing, China). All chemicals were used as received without further purification.

### Preparation of graphene oxide sheets

Ultrathin graphene oxide (GO) sheets were obtained by chemical treatment of exfoliated graphite powders according to the modified Hummers’ method^46,47^. Typically, 3 g of graphite powers and 1.5 g of NaNO_3_ were added into 70 mL of 98% H_2_SO_4_ and stirred in an ice-water bath. Subsequently, 9 g of KMnO_4_ was added slowly. After 2 h, the obtained mixture was transferred into the water bath and kept at 35 °C for 30 min. Next, 150 mL of deionized water was added gradually, meanwhile, the temperature was maintained lower than 50 °C by controlling the speed of dripping water. After that, the water bath was raised up to 95 °C and kept constant for 30 min. Then, 15 mL of 30% H_2_O_2_ solution and 50 mL of warm deionized water were dropped into the mixture to obtain the diluted bright yellow suspension. The resulting suspension was cleaned using HCl aqueous (1:10) solution and deionized water. Finally, the obtained GO solution was carefully diluted into 2 mg/mL using deionized water.

### Preparation of porous AC materials

Waste fiberboard materials were supplied by Beijing Jiahekailai Furniture and Design Company, which contained 12wt % urea-formaldehyde resin adhesive. Firstly, the materials were carbonized in a high-purity nitrogen atmosphere and heated to the temperature of 500 °C at the heating rate of 10 °C/min, then maintained for 60 min. After carbonization, the obtained materials were mixed with KOH at the mass ratio of 1:3 and further activated at the temperature of 750 °C for 60 min in oven. Finally, the activated ACs were washed and filtered using deionized water and 1 M HCl solution respectively until to neutral pH. Then dried at 105 °C for 8 h and stored for subsequent use^48,49^.

### Preparation of GN/AC flexible films

The AC powder and acetylene black with a mass ratio of 9:1 were mixed and dispersed in N,N-dimethylformamide (DMF) under ultrasonic vibration to produce a homogeneous dispersion of 0.5 mg/mL. Based on the premise of ensuring the formation of flexible film, AC was used to replace GN with the maximum limit. 5 mL of GO suspension was mixed with 40 mL of AC dispersion, corresponding 5 mL of DMF was added in Erlenmeyer flask to ensure that the volume ratio of water to DMF was 1:9, which was advantageous to improve the dispersion of GO^50^. Then the mixture solution was sealed with preservative film and under ultrasonic vibration for 2 h. Subsequently, a small quantity of 80% hydrazine hydrate was added in the resulting homogeneous dispersion. The weight ratio of hydrazine hydrate to GO was about 7:10^51^. After being vigorously shaken or stirred for 5 min, the Erlenmeyer flask was put in a water bath (~95 °C) for 3 h. Finally, the mixture solution was vacuum-filtrated on the organic microporous membrane filter at constant pressure.

### Preparation of GN/AC/MnO_2_ composite electrodes

In order to prepare the GN/AC/MnO_2_ composite electrodes, the three-dimensional GN/AC flexible films were cut into 1 × 1.5 cm pieces and an electro-deposition process was performed in a three-electrode system with GN/AC substrate as the working electrode, platinum plate electrode as counter electrode, and calomel electrode as reference electrode. The electrolyte contained 0.5 M Mn(CH_3_COO)_2_·4H_2_O and 0.5 M Na_2_SO_4_.Anodic constant current deposition method (at the current density of 8 mA/cm^2^) was applied to deposit MnO_2_ on the working electrode. The GN/AC/MnO_2_ composite electrodes at different reaction times were donated as GN/AC/MnO_2_-x, where x is the reaction time (s). The weight of MnO_2_ in GN/AC/MnO_2_ electrode was calculated by weighing before and after the GN/AC loading MnO_2_ (Mettler ToledoXP56, resolution of 1 μg).

### Assembly of flexible solid-state SCs

Two pieces of the flexible electrode films of GN/AC/MnO_2_ were immersed in the hot Na_2_SO_4_/Polyvinyl alcohol (PVA) gel electrolyte (3 g Na_2_SO_4_ and 3 g PVA were added into 30 mL deionized water) for 60 min, and subsequently picked out for air-drying to evaporate the residual water. Then, they were assembled in parallel and packaged together by Ni foam. Finally, the device was pressed under a pressure of ∼1 MPa for 30 min, which can make them adhere tightly and facilitate the polymer gel electrolyte penetrating into them.

### Characterizations

The microscopic morphologies of the composites were characterized by scanning electron microscopy (SEM, JEOL JSM-7001F), transmission electron microscopy (TEM, JEM-1010) and field emission scanning electron microscopy (FESEM, SU8010). X-ray diffraction (XRD) was used to analyze the crystal structure of the composites using a Bruker D8 diffractometer with Cu Kα radiation. Raman spectra were collected by a LabRAM HR Evolution Raman spectroscope using a 532 nm laser source. X-ray photoelectron spectroscopy (XPS) characterizations were conducted using an Axis Ultra DLD X-ray photoelectron spectroscopy.

### Electrochemical measurements

Electrochemical performances of the composites were evaluated by a CHI 660D electrochemical workstation using a three-electrode system, in which platinum plate electrode and saturated calomel electrode were used as counter electrode and reference electrode in an 1 M Na_2_SO_4_ aqueous solution, respectively. The GN/AC/MnO_2_ composite electrode was used as the working electrode. Cyclic voltammetry (CV), galvanostatic charge/discharge (GCD), impedance spectroscopy (EIS) and cycling stability were measured systematically. The areal or volumetric specific capacitance (${C}_{s,{electrode}}$ or ${C}_{V,{cell}}$) of single electrode or flexible SCs were calculated by the CV and GCD curves using the following equations:

1 $${C}_{s,{electrode}}=\frac{1}{{S}_{{electrode}}v\triangle {\rm{V}}}\int I(V){dV}$$

2 $${C}_{s,{electrode}}=\frac{I\triangle {\rm{t}}}{{S}_{{electrode}}\triangle {\rm{V}}}$$

3 $${C}_{V,{cell}}=\frac{I\triangle {\rm{t}}}{{{\rm{V}}}_{{cell}}\triangle {\rm{V}}}$$

The energy density (${E}_{V}$, mWh/cm^3^) and power density (${P}_{V}$, W/cm^3^) was calculated by the following equations:

4 $${E}_{V}=\frac{1}{2}{C}_{V,{cell}}{\triangle {\rm{V}}}^{2}$$

5 $${P}_{V}=\frac{{E}_{V}}{\triangle {\rm{t}}}$$

where ${I}$ (A) is the charge/discharge current, $v$ (mV/s) is the scan rate, $\triangle {\rm{t}}$ (s) is the discharging time, ${S}_{{electrode}}$ (cm^2^) is the area of single electrode, ${{\rm{V}}}_{{cell}}$ (cm^3^) is the volume of flexible SCs, and $\triangle {\rm{V}}$ is the potential window.
